# Anti-Inflammatory and Anticancer Properties of Birch Bark-Derived Betulin: Recent Developments

**DOI:** 10.3390/plants10122663

**Published:** 2021-12-03

**Authors:** Hardeep Singh Tuli, Katrin Sak, Dhruv Sanjay Gupta, Ginpreet Kaur, Diwakar Aggarwal, Nidarshana Chaturvedi Parashar, Renuka Choudhary, Mukerrem Betul Yerer, Jagjit Kaur, Manoj Kumar, Vivek Kumar Garg, Gautam Sethi

**Affiliations:** 1 Department of Biotechnology, Maharishi Markandeshwar (Deemed to be University), Mullana, Ambala 133207, Haryana, India; diwakaraggarwal@yahoo.co.in (D.A.); nidarshanachaturvedi@gmail.com (N.C.P.); renuka.ndri@gmail.com (R.C.); 2 NGO Praeventio, 50407 Tartu, Estonia; katrin.sak.001@mail.ee; 3 Shobhaben Pratapbhai Patel School of Pharmacy and Technology Management, SVKM’s NMIMS, Mumbai 40056, Maharashtra, India; dhruvg2507@gmail.com (D.S.G.); ginpreet.kaur@nmims.edu (G.K.); 4 Department of Pharmacology, Faculty of Pharmacy, Erciyes University, Kayseri 38039, Turkey; eczbetul@yahoo.com; 5 ARC Centre of Excellence in Nanoscale Biophotonics (CNBP), Graduate School of Biomedical Engineering, Faculty of Engineering, The University of New South Wales, Sydney 2052, Australia; 1990jagjit@gmail.com; 6 Department of Chemistry, Maharishi Markandeshwar University, Sadopur 134007, Haryana, India; manojraju27@gmail.com; 7 Department of Medical Laboratory Technology, University Institute of Applied Health Sciences, Chandigarh University, Gharuan, Mohali 140413, Punjab, India; garg.vivek85@gmail.com; 8 Department of Pharmacology, Yong Loo Lin School of Medicine, National University of Singapore, Singapore 117600, Singapore

**Keywords:** birch bark, betulin, inflammation, cancer, NF-κB, Nrf2, nanocarriers

## Abstract

Birch tree bark-derived betulin has attracted scientific interest already for several centuries, being one of the first natural products identified from plants. However, the cellular events regulated by betulin and precise molecular mechanisms under these processes have been begun to be understood only recently. Today, we know that betulin can exert important anticancer activities through modulation of diverse cellular pathways. In this review article, betulin-regulated molecular signaling is unraveled and presented with a special focus on its participation in anti-inflammatory processes, especially by modulating nuclear factor-κB (NF-κB), prostaglandin/COX, and nuclear factor erythroid2-related factor 2 (Nrf2)-mediated cascades. By regulating these diverse pathways, betulin can not only affect the development and progression of different cancers, but also enhance the antitumor action of traditional therapeutic modalities. It is expected that by overcoming the low bioavailability of betulin by encapsulating it into nanocarriers, this promising natural compound may provide novel possibilities for targeting inflammation-related cancers.

## 1. Introduction

Natural products have been a highly attractive source for different pharmacological substances and therapeutic agents for several decades, particularly for infectious diseases at 75% and cancer at 60% of new drugs are originated from different natural sources [1,2,3,4]. In fact, several well-known anticancer drugs have been initially isolated from plants with at least nine plant-derived compounds approved for the use in clinical settings since 1961 [5]. These substances include vinblastine and vincristine from the *Madagascar periwinkle* plant, paclitaxel from the bark of the Pacific yew (*Taxus brevifolia*) tree, podophyllotoxin from the roots of the mayapple plant (family *Berberidaceae*), and camptothecin from certain angiosperms [5]. Considering that the incidence rate of new cancer cases is expected to continuously increase each year [6,7,8,9,10,11,12,13,14] and many types of malignancies are still remained incurable, further devoted investigation into phytochemicals can provide potential novel leads for developing new anticancer drugs with higher efficiency and broader safety profile [15,16,17,18].

Betulin, a naturally occurring triterpene, is commonly derived from the bark of birch trees *Betula* L. [19]. This compound was first isolated as a pure chemical substance already in 1788, being one of the first natural products identified from plants [20]. Besides giving the tree its white color and thereby protecting birches from midwinter overheating by the sun [21], a number of recent studies have shown that betulin is biologically active also in human beings, particularly against development of different tumors [19]. Although the exact molecular mechanisms underlying anticancer action of betulin have still remained to be unraveled, they have been largely related to anti-inflammatory activities of this phytochemical [22]. In this way, the role of betulin against inflammation-associated malignancies has been often demonstrated, describing its growth inhibitory and apoptosis-inducing effects in a wide spectrum of human malignancies, including colorectal, gastric, liver, lung, breast, ovarian, cervical and prostate cancer cells [23]. In this review article, the current preclinical knowledge about anti- inflammatory and anticancer properties of betulin is compiled and systematically presented, highlighting besides the bottlenecks also the potential solutions to move on to clinical trials.

## 2. Chemistry of Betulin

**Figure 1:**
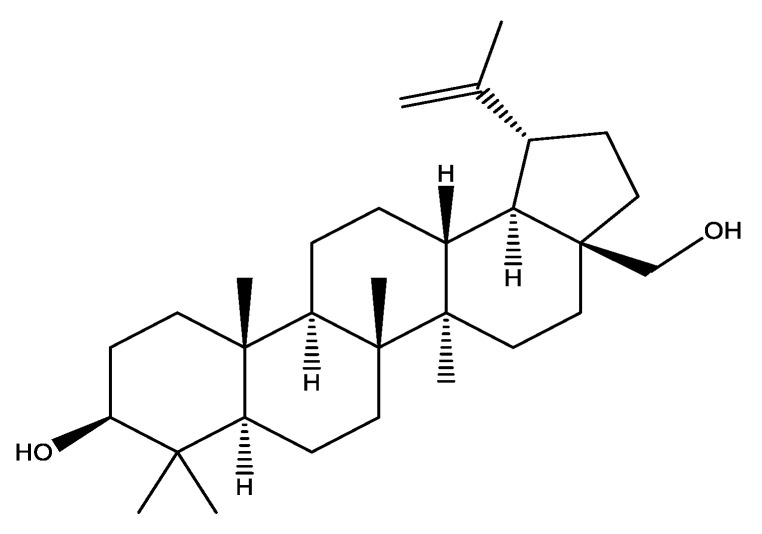
Structure formula of betulin.

Chemically, betulin is a pentacyclic triterpenoid which is also known as betulinic alcohol (Figure 1) obtained from bark of white birch species found in northern latitude of world including Alaska, Canada, Europe, Russia and Asia [24].

**Figure 2:**
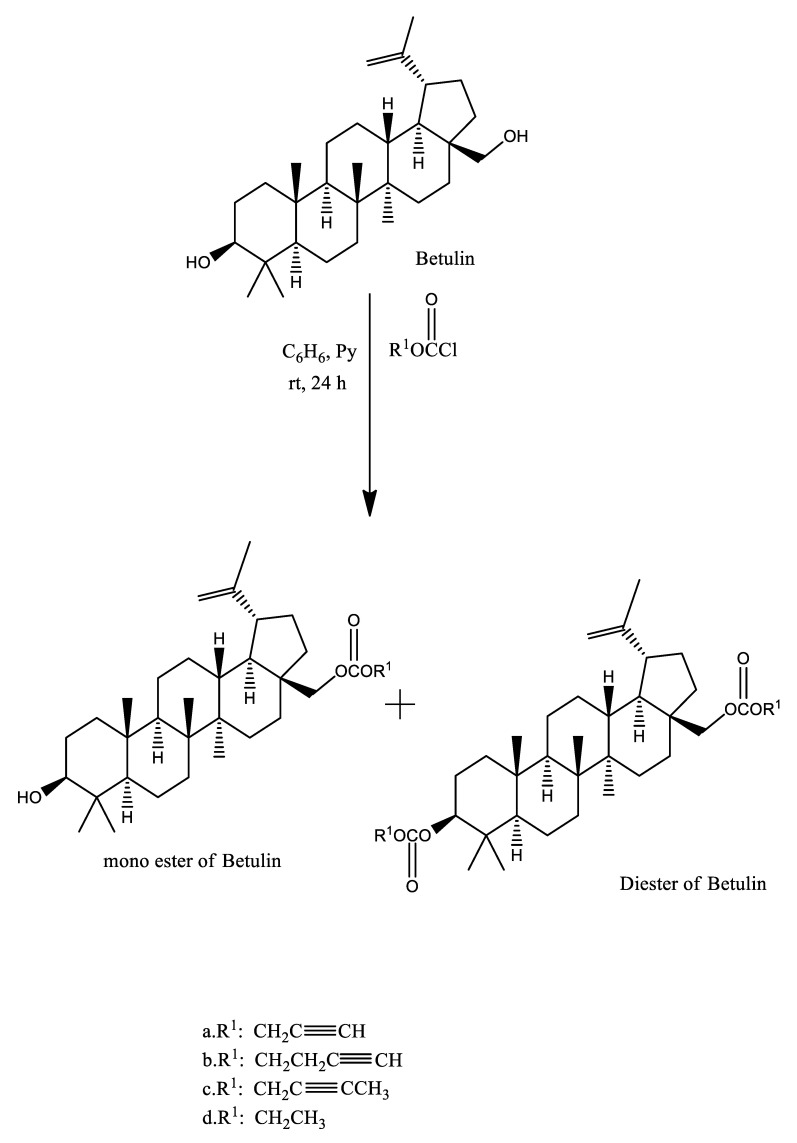
Synthesis of acetylenic derivatives of Betulin.

Chemical modifications in betulin can be easily accomplished at positions C–28, C–3, and C–20 [25]. Many reports have been found in literature disclosing the pathway for the synthesis of betulin’s derivatives. Presence of the high content of betulin in white birch bark (up to 30%) makes it suitable for the synthesis of biologically active derivatives of Betulin. Boryczka et al. in 2013 reported the synthesis of new interesting acetylenic derivatives of Betulin by treating a mixture of Betulin and pyridine in dry benzene [26] with propargyl chloroformate (a), 2-butyn-1-yl chloroformate (b), 3-butyn-1-yl chloroformate (c), ethyl chloroformate (d) respectively in dry benzene (Figure 2).

**Figure 3:**
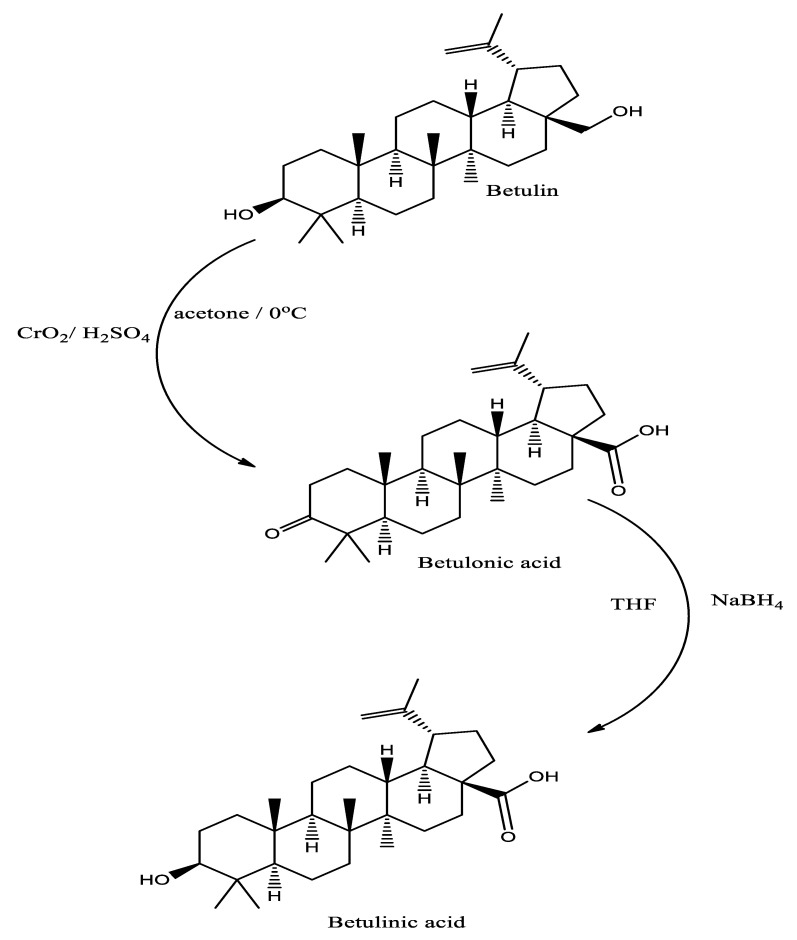
Synthesis of betulinic acid from Betulin by Jones oxidation followed by reduction.

Betulinic acid is an important natural derivative which is formed by the oxidative reactions of betulin. Methanolic and ethanolic extractions of various plant parts are found to possess significant amount of betulinic acid [27]. Betulinic acid can also be synthesized from betulin by a two-step chemical reaction. In first step oxidation of the C3 and C28 hydroxyls occurred followed by the reduction of betulonic acid (Figure 3) by using sodium borohydride [28].

## 3. Absorption, Metabolic Conversion, and Bioavailability of Betulin

Betulin exhibits enormous pharmacological potential [22,23,24,25], owing to its relatively small size and specific cytotoxic actions against tumor cells. This has encouraged research on the molecule, aiming to highlight its advantage as compared to conventional therapeutic agents.

Experimental studies carried out on human and rat hepatic microsomes and cytosol indicated that two major biotransformation pathways for betulin are glucuronidation and sulfonation [29]. The data obtained from studies in rat models showed that hUGT1A3 and 1A4 were the main hepatic enzymes responsible for the formation of possibly a C3- hydroxyl betulin glucuronide, while hSULT2A1 (responsible for the conversion of betulin into betulin sulphate I and II) was the main isoform involved in sulfonation. In human systems, glucuronidation occurs hepatically and extra-hepatically (in the gastrointestinal tract), and the same enzymes as rat models being predominant in catalyzing the reactions. One betulin glucuronide and two betulin sulfates were yielded at the end of the metabolism process [29].

In vivo studies have shown that the carbon-carbon double bond and hydroxy functional group are the main metabolism sites for betulin. The compound undergoes demethylation, dehydroxylation, deoxidization, dehydration as a part of the phase-1 metabolic reactions, followed by conjugation reactions with cysteine, sulfate, taurine and N-acetylcysteine as a part of phase-2 metabolism. Metabolites are obtained at each stage, as a product of the reactions. A total of 62 metabolites of betulin have been studied, a majority of which are obtained from phase-1 biotransformation [30].

An experimental study has demonstrated the modulation of mitochondrial membranes, in case of colon and prostate cancer, which enhances the uptake of membrane proteins and expression of cytochrome c oxidase. Anti-cancer agents such as betulin may exert therapeutic effects by targeting the modified membranes, owing to the high affinity of betulinic acid to the lipid monolayers present on the membranes. This mechanism may inhibit the growth and multiplication of cancerous cells [31].

Betulin and its product of oxidation, betulinic acid show poor aqueous solubility owing to their structure. Hence, various derivatives, such as amino acid esters, have been synthesized to improve bioavailability and delivery to target tissues [32]. Additionally, various 3-modified derivatives have been synthesized, which show promising ADME parameters and are more hydrophilic. These derivatives showed hydrogen bond acceptor (HBA) and hydrogen bond donor (HBD) values lying in the required range, along with low TPSA values, which facilitate delivery across the blood-brain barrier. As these derivatives show good transport properties, they may be harnessed for the treatment of neoplasms of the central nervous system [33]. These derivatives are synthesized with the purpose of enhancing the therapeutic potentials of betulin.

## 4. Anti-Inflammatory Mechanisms Involved in Anticancer Action of Betulin

### 4.1. NF-kB-Mediated Signaling

**Figure 4:**
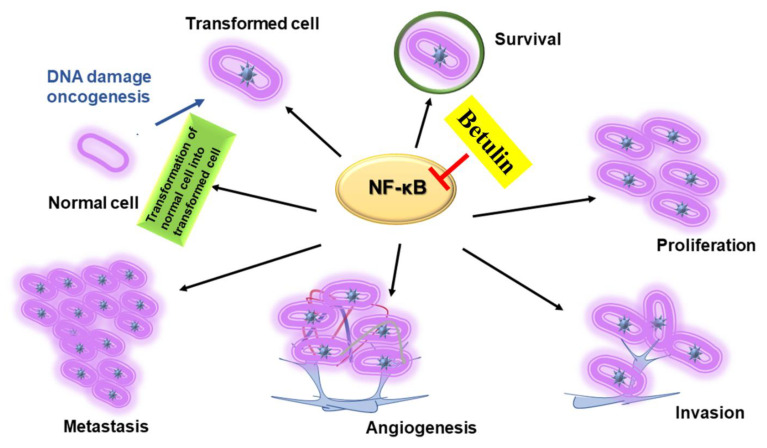
Role of betulin in inhibiting NF-ĸB mediated inflammatory mechanisms involved in the transformation of normal cells to cancer cells.

NF-κB is known to induce the expression of a diverse range of inflammatory genes that are further found to modulate the transcriptional rate of various cytokines and chemokines (interferons, interleukins, lymphokines, tumour necrosis factor) [34,35,36]. In addition, NF-κB displays a promising role in modulating the cancer cell survival, and differentiation signaling cascade [37,38,39]. Currently a vast amount of research is carried on cancer treatment therapies [40,41] however, there are still some issues to be solved such as chronic inflammatory micro environment of tumor and high mortality rates. Therefore, the researchers have concentrated on developing the anti-inflammatory agents that could successfully treat cancer without causing any or minimal side-effects [42,43,44,45]. One answer to this problem is to use of the anti-inflammatory phytochemicals such as betulin for the cancer treatment. Betulin has proven to possess anti-cancerous and anti-inflammatory properties against pancreatic, gastric, lung, ovarian [46], melanoma cells [47], and nervous system carcinomas [48]. In addition, its cytotoxic effect on normal cells was lesser as compared towards the cancer cells [49,50]. It affects the expression of NF-κB and triggers a diverse range of cellular mechanisms like cell-cycle arrest, cell viability inhibition, apoptosis induction, invasion/migration inhibition, and anti-angiogenesis (Figure 4). The tumors are formed in the body when the dynamic balance between the cell death and cell proliferation is disturbed, and excess of cell proliferation is caused. Therefore, apoptosis induction of the affected cells could be a good choice of treatment of the cancerous cells.

Apoptosis or programmed cell death is the mechanism of the cells to remove the superfluous, damaged, and defective cells [11,51] through release of cytochrome c and activation of caspase-9 or activation of caspase-8 via pro-apoptotic receptors [52,53,54]. The cancerous cells overexpress the anti-apoptotic proteins (Bcl-2 and XIAP) and betulin targets them to express its anti-cancerous properties [55] by generation of reactive oxygen species (ROS). In a study, it was found that the gastric cancer SGC7901, MDA-MB-231 breast cancer, and colon carcinoma (Caco 2) cell growth was inhibited by betulin as it triggered mitochondrial release of cytochrome c, mitochondrial translocation of Bak, and Bax, and down-regulation of NF-κB p50 and 65, IKKα and β, ICAM-1 and bcl-2 [56,57,58]. Therefore, betulin can be used as an anti-cancerous agent for various types of cancers.

### 4.2. Prostaglandin/COX-2-Regulated Inflammatory Events

For numerous inflammatory pathways, the arachidonic acid (AA) acts as one of the most important metabolic precursor [59,60]. The membrane bound AA cleaves from the phospholipids after the activation of phospholipase A2 (PLA2) by external and internal factors which gets available to various inflammatory events such as lipoxygenase, cytochrome P-450 monooxygenase and cyclooxygenase pathway [61]. In mammals, the most comprehensively studied inflammatory pathway is cyclooxygenase pathway, which begins with AA conversion to PGH_2_ (substrate for prostaglandin) due to the action of prostaglandin G/H synthase commonly referred as cyclooxygenases [62,63]. The COX-1 and COX-2 are the isoenzymes of cyclooxygenase enzymes. COX-1 acts as a housekeeping enzyme, as it is constitutive in nature and expressed in various parts of the body. In addition to this, it also carries out numerous physiological functions. Studies on mice revealed that COX-1 also plays a crucial role in development and progression of inflammation [64,65,66,67].

On the other side, COX-2 is mainly induced in response to various endogenous and exogenous stimulus such as cytokines (tumor necrosis factor α (TNF-α), interleukins (IL-1 and IL-6), tumor promoters (v-src, v-Ha-ras, and Wnt)) and stress [68,69]. It is mainly responsible for maintenance of inflammatory event after the initiation of inflammatory acute phase with COX-1 [70]. Although COX-2 shows the structural similarity with COX-1, its enzymatic activity pattern is quite different due to prostaglandin-endoperoxide synthase 2 (PTGS-2) gene. After the induction of COX-2, there is excessive production of PGE_2_ along with other prostaglandins, which increases the vascular permeability and lowers the pain threshold. The physiological functions like blood pressure and immune response are maintained by PGE_2_, but in some pathological conditions more than 10-fold increase in the level of PGE_2_ concentration leads to serious complications [71]. Controlled level of COX-2 enzyme production plays a crucial role in the physiological protective response to tissue injury. However, if uncontrolled enzyme production occurs, it can promote angiogenesis and tumor invasiveness and ultimately causes inflammatory-induced carcinogenesis [72,73,74,75,76].

**Figure 5:**
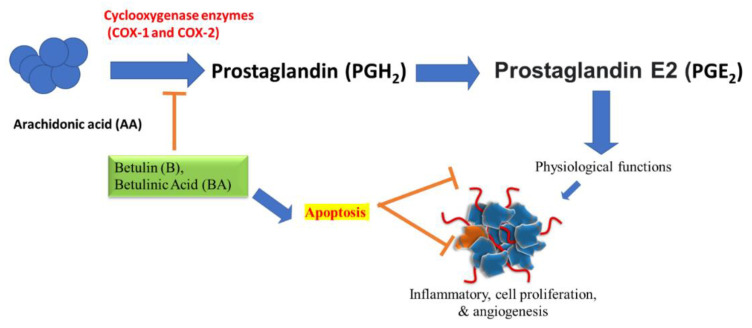
Action of Betulin (B) and Betulinic Acid (BA) on cyclooxygenase enzymes (Cox-1 and Cox-2) which convert the arachidonic acid to prostaglandins. Inhibition of prostaglandin PGE_2_ derived from Cox-2 by blocking its pathway through Betulin (B) and Betulinic Acid (BA) leads to inhibition of angiogenesis, proliferative invasion.

For decades, traditional methods using natural products were used as medicines for treating numerous diseases [77]. There are evidences of using these as remedies from pre historic times for all sorts of inflammatory diseases. Since, COX-2 is responsible for inflammatory events through PGE_2_ production and its uncontrolled level can cause carcinogenesis. Therefore, it is widely accepted that natural product having potential to inhibit COX-2 and PGE_2_ expression, will exhibit anti-inflammatory and anti-cancerous activities. Numerous triterpenoids such as betulin (B) and betulinic acid (BA) (the oxidation product of Betulin) isolated from botanical sources play an important role in inflammation reduction and exhibit anti-cancerous properties by targeting COX-2 and PGE_2_. These can induce anti-inflammatory, tumor-differentiating, proliferation-arresting, and apoptotic effects based on the usage of their dose administered [78]. Recent study on immunopharmacological activity of betulin revealed that it has a potential use in inflammation-associated carcinogenesis [22]. The derivatives of betulin can also inhibit IFN-γ and modulate COX-2 expression [79]. Previous studies reported that betulinic acid can inhibit the cyclooxygenase pathway by reducing the synthesis of prostaglandins (PGE_2_) and attenuate the inflammation in response of stimuli [57,80,81]. Study on betulinic acid isolated from the *Dillenia serrata* also revealed the same that betulinic acid can modulate the activity of COX-2 and inhibit the PGE_2_ release [82]. This COX-2-mediated inhibition of prostaglandin by betulin and betulinic acid controls the cell proliferation, angiogenesis, invasion and metastasis [83]. Collectively, based on evidence in the literature it can be stated that betulin and its oxidation product betulinic acid induces its potent anti-inflammatory and anti-cancerous effects by blocking COX-2-mediated NF-κB pathway mechanisms [78,79,80,81]. They can control the inflammation-induced cancer by inhibiting proliferation, invasion, metastasis, angiogenesis and inducing apoptosis, but more clinical investigations are required in order to support the proposed COX-2 inhibitory mechanism by betulin and betulinic acid (Figure 5).

### 4.3. Nrf2-Associated Signaling

**Figure 6:**
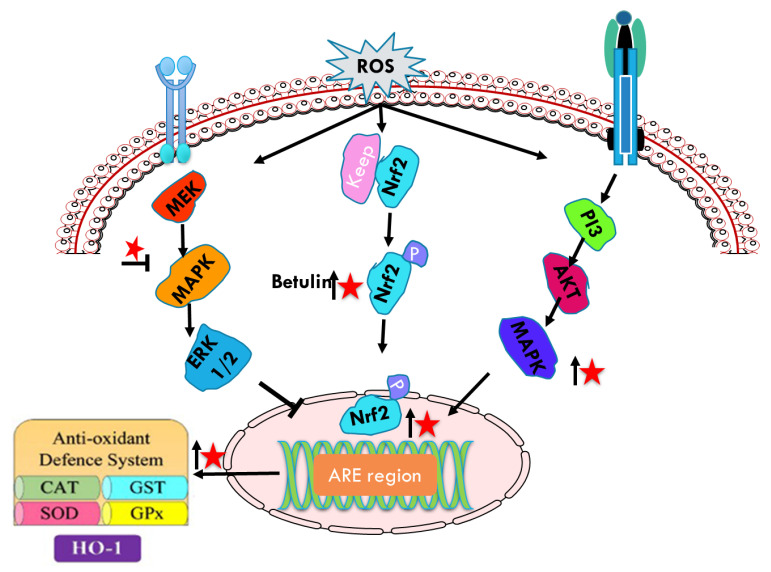
Schematic representations of betulin (represented as red star) mediated modulation (upregulation ↑, downregulation **↓**) of Nrf 2 phosphorylation and anti-oxidant defense system.

Betulin has been associated with its antiinflammatory effect over different cellular mechanisms including the nuclear factor erythroid 2-related factor 2 (Nrf2), a critical transcriptional activator for antioxidative responses (Figure 6). Nrf2 is a transcription factor that regulates an adaptive cellular defense response to oxidative stress and inflammation [84,85]. It plays a crucial role in cellular redox homeostasis coordinating the induction of over 250 genes, including those encoding antioxidant and phase 2 detoxifying enzymes and related proteins, such as NADPH, quinine oxidoreductase 1 (NQO1), heme oxygenase-1 (HO-1), γ-glutamyl cysteine synthetase catalytic subunit (GCLC) and modifier subunit (GCLM) [86].

Phosphorylation of Nrf2 at serine and threonine residues by upstream kinases, such as protein kinase C, phosphatidylinositol-3-kinase/Akt (PI3K/Akt), and mitogen-activated protein kinase (MAPK), facilitates the release of Nrf2 from Keap1, a repressor molecule that facilitates Nrf2 ubiquitination [87]. Nrf2 phosphorylation regulating the cellular responses to oxidative stress and inflammation is also regulated by AMP-activated protein kinase (AMPK), a heterotrimeric serine/threonine kinase [88]. Activated Nrf2 quickly translocates from the cytoplasm into the nucleus to regulate gene expression. Nrf2 is anchored within the cytoplasm by Kelch-like-ECH-associated protein 1 (Keap1) before ubiquitarian. AMPK is also associated with PI3K/Akt pathway that has been shown to be regulated with AMPK. Furthermore, AMPK increases the phosphorylation of glycogen synthase kinase 3 beta (GSK3β) inhibition [89]. Nrf2 signaling has been implicated as an important target for averting DMBA-induced mammary cancer via augmented expression of MAPKs, Keap1, ARNT, AhR, and CYP1A1 [90]. Therefore, strong antioxidant behavior of betulin by Nrf2 mediated MAPKs oxidative stress could be considered to inhibit cancer proliferation. Ci et al. [91] has shown that betulin increased Nrf2-targeted antioxidant enzymes, in a dose and time dependent manner in LPS and endotoxin induced inflammatory responses in vitro and in vivo. Treatment with betulin increased Nrf2 translocation from cytoplasm to nucleus and downregulated the expression of the Keap1 protein in a dose-dependent manner. Furthermore, betulin attenuated LPS-induced inflammatory mediators (iNOS and COX-2) and MAPK inflammatory signaling pathway upregulating the HO-1 and NQO1, and downregulating the iNOS and COX-2 revealing that its anti-inflammatory effect is strongly coordinated with Nrf2 signaling pathways. Furthermore, betulin pretreatment reduced the increased levels of JNK, ERK, p38 and AKT phosphorylation in LPS induced macrophages.

Activation of Nrf2 by triterpenoids induces the expression of phase 2 detoxifying and antioxidant enzymes such as NQO1 and HO-1, known enzymes which can protect cells or tissues against various toxic metabolites [92]. Bai et al. [93] has revealed that betulinic acid attenuates impairments of aortic contraction and relaxation in LPS-challenged rats by activating Nrf2-regulated anti-oxidative pathways.

Nrf2-mediated anti-inflammatory response is thought to be ROS-dependent, however a direct inhibitory effect of Nrf2 on the recruitment of RNA polymerase II, preventing the transcription of genes coding for the proinflammatory cytokines IL-1β, IL-6 [94,95,96], which is also important for the viral infections including Covid-19 [97,98]. Activation of Nrf2 signaling pathway in phagocytic cells improved their anti-viral [99], and anti-bacterial functions [100]. Furthermore it has been noted that in macrophages regulating the Nrf2 mechanisms in bacterial infections is very important to control the inflammation [101]. The most common Nrf2 nutrients has been listed by Iddir et al. [102] including flavonoids and terpenoids [103] and betulin has also been shown to act through Nrf2 signaling which deserves further investigation on Covid-19.

## 5. Betulin as a Treatment Strategy for Cancer

To design effective cancer treatment strategy, it is essential to understand the interactions of natural bioactive molecules with the recognized cellular targets. Several anti-cancer agents have been known to mediate both intrinsic (mitochondrial) as well as extrinsic (Fas/FasL) apoptotic cell death in cancer cells [104,105]. Previous studies have suggested the role of bioactive natural molecules to arrest cell cycle by regulating the expression of cyclin-dependent kinases (CDKs) [106,107]. In addition, the expression of metastatic as well angiogenesis proteins including matrix metalloproteinases (MMPs) and VEGF have also been down-regulated by the action of such bioactive metabolites [108,109]. Furthermore, the anti-tumor aspect of natural metabolites can be correlated with their inhibitory effects on various inflammatory mediators (IL-6, IL-8, IFN-γ, iNOS, COX-2, and TNF-α) [110,111,112]. Therefore, exploring the mechanistic insight of bioactive molecules will help us to understand the biology of cancer and to investigate novel anti-cancer strategies in the near future [113]. For instance, researchers have investigated the interaction of tumor cells with their microenvironment to develop promising anti-cancer strategy. Modulations of expression/activity of TNF-β-, NF-κB, MMP-9, CXCR4, Ki-67, β1-integrin, and caspase-3 could be a promising strategy for tumor control. Therefore, suppression of proinflammatory molecules by using natural agents can inhibit the cancer growth, survival, and metastasis [114,115].

### 5.1. Co-Effects of Betulin with Standard Anticancer Therapies

Secondary metabolites or natural compounds found ubiquitously distributed in different plant types have been documented to potentiate standard chemo-preventive measures used for cancer treatment [116,117]. Such combinatorial or synergistic approaches exhibit remarkable efficacy in cancer therapy due to their multi-targeted actions, minimum side-effects with little or no drug resistance and lack of considerable toxicity [118,119,120,121,122]. Betulin when used in combination with a gamma-cyclodextrin derivative in melanoma B164A5 cells, the combinatorial therapy was found to reduce the cell proliferation, and induced differentiation and cell death [123]. Further, combination strategy using betulinic acid and its derivatives in combination with radiation therapy on human malignant glioma cell lines has shown slightly enhanced effects on the radiosensitivity of malignant glioma cells [124]. In addition, few studies have shown to have an additive effect of the compound in combination with irradiation on growth inhibition in melanoma [125] and head and neck squamous cell carcinoma (HNSCC) cell lines [126]. Moreover, in one of the studies, the efficacy of 5-fluorouracil (5-FU) and betulinic acid (BA) combination on ovarian carcinoma cells was studied and the results demonstrated increased sub-G1 cell population, increased rate of cell apoptosis and morphological changes in mitochondrial membrane. Therefore, the combinatorial therapy was found to be a promising strategy for the treatment of ovarian carcinoma [127]. Furthermore, the study was conducted to explore the interactions between the natural compound and tumor necrosis factor-related apoptosis-inducing ligand of APO2, also known as TRAIL, in liver cancer cells and a synergistic effect of betulinic acid and APO2 combination on apoptosis induction in liver cancer cells was observed [128]. Additionally, the compound also showed synergistic effects with taxol to induce breast cancer cells G2/M checkpoint arrest and apoptosis induction, but had little cytotoxicity effects on normal mammary epithelial cells [129]. Combination treatment of the compound with ginsenoside Rh2 (G-Rh2) synergistically induced apoptosis in human cervical adenocarcinoma (HeLa), human lung cancer A549, and human hepatoma HepG2 cells by enhancing cleavage of caspase-8 and Bid [130]. Likewise, studies have also shown that betulinic acid along with other triterpenes, especially Japanese apricot extract, are effective supplements for increasing the chemotherapeutic effect of 5-fluorouracil on esophageal cancer [131]. In conclusion, betulin and its derivatives like betulinic acid could prove to be promising treatment agents in various cancer types and a combination of the natural compound with different chemotherapeutic drugs seems to be beneficial.

### 5.2. Role of Nanotechnology in Delivery of Betulin to Target Tissues

Despite poor aqueous solubility, triterpenoids such as betulin have gained interest in the arena of nanotechnology on account of their potent cytotoxic properties. Formulating these compounds as nanopharmaceuticals additionally helps to enhance systemic bioavailability and stability of such phytoconstituents [132,133]. Betulin was first encapsulated in liposomes by Mullauer et al., that could be used for the amelioration of colon and lung cancer tumors [134]. More recently, Liu et al., formulated polyethylene-glycol modified liposomes of Betulinic acid, which showed promising in vivo results [135].

The formulation of liposomes has a two-fold impact: enhancement of solubility as well as increasing the affinity of the agent to tumor cells, which enhances the permeation, and thereby efficacy of the molecule. Liposomes containing betulinic acid and a biosurfactant mannosylerythritol lipid-A (MEL-A) have been observed to trigger early-stage apoptosis of HepG2 cells, which in turn blocks cell division, thereby arresting tumor growth [136]. In addition to this, micellar systems have been formulated to improve the delivery of betulin to target cells. Loading co-polymeric Soluplus micelles with betulinic acid has been seen to inhibit angiogenesis, DNA replication and tumor growth in vivo, specifically for breast cancer cells [137].

In relation to nanoemulsions, Dehelean et al., formulated a nanoemulsion of betulinic acid by high-pressure homogenization, using flax-seed oil as the oil phase [138]. The anti-neoplastic effects of betulinic acid were assessed in vivo by Tan et al., using nanoparticles of betulinic acid, establishing that the magnetic nanoparticles may facilitate improved entry of the drug into cells [139]. betulinic acid was also incorporated into a γ-cyclodextrin complex, thus allowing studies of betulinic acid delivery using cyclodextrin inclusion complexes [140].

In a study, liquid crystalline nanoparticles of betulinic acid were formulated, helping to expand its therapeutic potential. The objective of such experiments is the formulation of theranostics, for drug delivery to specific, targeted tissues. Betulinic acid, in combination with manganese, was administered to mammalian breast cancer cell lines, as they demonstrate a synergistic effect. The formulation passed the biosafety test, carried out on embryonic hepatic cell lines, thus establishing its safety in biological systems. The apoptosis of MDA-MB-231 cells was seen by the onset of oxidative stress, as well as the exertion of an anti-inflammatory action [141]. In an attempt to improve the oral bioavailability of betulinic acid, incorporation into poly(lactic-co-glycolic acid) (PLGA) has been reported to exert a preventive action against hepatocellular carcinoma (HCC) [142].

Silver-based nanoparticles have been used as drug-delivery agents owing to their surface properties and mild cytotoxic properties, which make them potent anti-cancer agents. Based on data obtained from pre-clinical research, the usage of betulin in conjunction with silver nanocolloids (uncoated as well as PEG-coated) has shown efficacy in inhibiting the proliferation of HepG2 and A549 cells [143]. Cyclic β-glucans may be used for encapsulating betulinic acid, owing to their ability to form complexes and dose dependent antioxidant property. In-silico studies have demonstrated a synergistic interaction, thereby potentiating the anti-neoplastic properties of betulin [144].

These studies have yielded promising in vitro data, indicating the potential for usage of these nanotechnology systems in humans, as they help to combat some of the drawbacks of naturally derived triterpenes like betulin. Undertaking clinical trials would further improve the understanding of the therapeutic potentials and efficacy of betulin, thereby aiding management of neoplasms and various disorders with afflicted inflammatory pathways.

### 5.3. Safety Issue of Betulin

The most important aspect of any novel drug candidate is its safety on normal healthy tissues, allowing to elaborate the optimal dosage schemes with minimal adverse reactions [145,146]. Animal studies have shown no toxic symptoms and good tolerability of triterpene extract, being administered either intraperitoneally to rats (540 mg/kg for 28 days) or subcutaneously to beagle dogs (300 mg/kg/day for 28 days). Moreover, subcutaneous administration of betulin to male and female dogs resulted in a maximum plasma level of 325 ng/mL four weeks after treatment [147]. Also, betulin was shown to reveal no mutagenic activity by Salmonella/microsome assay, again proving its potential safety [148]. In addition, clinical trials with topical application of betulin-based Oleogel-S10 displayed well tolerability and safety of this treatment for patients with actinic keratoses [149], epidermolysis bullosa [150] or burn wounds [151]. Although all these data clearly show the general safety of betulin and encourage further pharmacological and pharmaceutical studies using this natural compound (Table 1 and Table 2), recently published results still demonstrate cytotoxicity of betulin in fish (BF-2) and murine fibroblasts (NIH/3T3) at doses similar to the IC_50_ values previously measured for malignant cells [152]. Therefore, further thorough research on the safety of betulin is needed, verifying the selectivity of cytotoxic action of this compound towards cancerous cells.

## 6. Conclusions

In this study, clear evidences are presented in favor of considering birch tree bark-derived betulin as a potential lead molecule for further development of anticancer agent. The anti-inflammatory properties of betulin would make it possible to apply this natural compound especially for the treatment of inflammation-related tumors. However, to reach this goal, the bottlenecks associated with low bioavailability should be solved first as well as the safety issues of this triterpene need to be enlightened. In this way, it is expected that betulin will represent “a long-known but newly discovered” phytochemical for the use in oncological field. The necessity for new anticancer drugs is obvious in view of several impediments related to the current treatment modalities, including acquired drug resistance and toxicities towards normal tissues. Therefore, identification and characterization of novel anticancer agents from naturally occurring products may lead to development of more efficient and safer cancer therapies in future, especially considering the steadily rising incidence rates of new cancer cases all over the world. In addition, chemical derivatizations or structural modifications of existing natural agents may also open new avenues in medicinal chemistry.

## Figures and Tables

**Table 1:** Anticancer effects of betulin and betulinic acid based on in vitro studies.

Type of Cancer	Cell Lines	Effects	Mechanisms	Concentration	References
Leukemia	Lucena 1 and K562	Blocking of the efflux mediated by P-gp	↑ restore sensitivity to doxorubicin in Lucena 1 cells, did not exhibit erythrocyte hemolysis	0.39–50 µM	[153]
Myeloma	RPMI 8226	Induces apoptosis	↓ proliferation, migration and invasion by tumor cells, ↓ bcl-2, ↑ bax, ↓ cyclin D1, No change in CREB phosphorylation,	0, 2.5, 5, 10 and 25 μM	[46]
Human T-cell leukemia	Jurkat E6.1	Induces apoptosis	↓ proliferation, migration and invasion by tumor cells, ↓ bcl-2, ↑ bax, ↓ cyclin D1, No change in CREB phosphorylation, Amounts of the CREB protein, and ERK1/2, Akt, CaMKII kinases remained unchanged	0, 2.5, 5, 10 and 25 μM	[46]
Glioma	T98G and C6	Induces apoptosis	↓ cell viability/survival and proliferation, ↓ % age of T98G cells in G1 phase, ↑ in cell number in S phase, significant activation of caspase 3	0.0–25 μM for EB5 or 0.0–50 μM for EB25/1	[154]
Osteosarcoma	HOS and MG-63	Induces autophagy↓↑	↑ LC 3-II, ↑ phospho-Akt (Ser473), ↓ activation of mTOR	0, 0.5, 1, 2, 4, 5, 10 and 20 μM	[155]
Medulloblastoma	TE671	Induces apoptosis	↓ proliferation, migration and invasion by tumor cells, ↓ bcl-2, ↑ bax, ↓ cyclin D1, No change in CREB phosphorylation, Amounts of the CREB protein, and ERK1/2, Akt, CaMKII kinases remained unchanged	0, 2.5, 5, 10 and 25 μM	[46]
Oral squamous	KB	Induced apoptosis	↓ cell proliferation, ↑ TUNEL+ cells in KB cells, ↑ caspase 3, ↑ caspase 9, ↑ Bax, ↓ Bcl-2, ↓ oxygen consumption rate, Induced a significant mitochondrial dysfunction, ↑ cell number in the G 0/G1 phase,	0, 12.5, 25, 50 and 100 μM	[156]
Thyroid	FTC 238	Induces apoptosis	↓ proliferation, migration and invasion by tumor cells, ↓ bcl-2, ↑ bax, ↓ cyclin D1, No change in CREB phosphorylation,	0, 2.5, 5, 10 and 25 μM	[46]
Melanoma	Colo-829	Induces apoptosis	↓ NQO1 protein, ↑ formation of superoxide, ↑ oxidative stress, ↑ TP53 ↑ CDKN1A genes, ↓ p53 protein	0.1 to 100 μg/mL	[157]
	C-32	Induces apoptosis	↓ transcription of the gene encoding the histone H3, ↓ NQO1 protein, ↑ formation of superoxide, ↑ oxidative stress, ↑ TP53 ↑ CDKN1A genes, ↑ BAX gene, ↓ BCL-2 gene, ↑ BAX/BCL-2 ratio, ↓ p53 protein	0.1 to 100 μg/mL	[157]
	Me-45	Induces apoptosis	↑ apoptotic nuclei, ↑ cytotoxicity towards malignant cells, ↑ apoptosis arte, ↑ pro-apoptotic effects, ↑ PARP-1, ↓ expression of caspase-3	0.75–100 µM	[32]
	B164A5 and B16F10	Induced apoptosis	↓ mitochondrial oxidoreductase, ↓ cell division rate, ↑ Bax, ↓ Bcl-2, ↑ IL-12p70 secretion, ↑ cleaved caspase 3, ↑ cleaved PARP	0, 40, 80, 120 and 160 μM	[158]
Epidermoid squamous	A431	Induces apoptosis	↑ apoptotic cells, ↑ Increased cytotoxicity for cancerous cells, ↑ PARP-1, ↓ amounts of caspase-3	0.75–100 μM	[159]
Breast	MDA-MB-231	Anti-angiogenic	↑ betulin uptake, ↓ cell viability of the cancer cells, ↑ in vitro cytotoxicity, ↑ mononucleated cells, ↓in binucleated cells	Nanosuspension of betulin equivalent to 5, 10, 25, 50, 100, 150 and 200 µM	[160]
	MDA-MB-231	Induces apoptosis	↓ cell size, ↑ shrinkage of the cytoplasm, ↓ NF-ĸB p65 and p50, ↓ IKK α and β, ↓ ICAM-1, ↓ bcl-2 expressions, significantly induced loss of mitochondrial transmembrane potential	0–50 μM	[58]
	MCF-7 and MDA-MB-231	Induces apoptosis	↓ histone H3, ↓ NQO1 protein, ↑ formation of superoxide, ↑ oxidative stress, ↑ TP53 ↑ CDKN1A genes, ↑ BAX gene, ↓ BCL-2 gene, ↑ BAX/BCL-2 ratio, ↓ p53 protein	0.1 to 100 μg/mL	[157]
	MDA-MB-231 and BT-549	Inhibited metastasis	↓ aerobicglycolysis, ↓reduction of lactate production, ↓ down regulation of aerobic glycolysis-related proteins, ↑ GRP78 overexpression, ↓ c-Myc-mediated glycolysis, ↓ MMP-2 and MMP-9, ↑ LDHB, ↑ PERK signaling, ↑ phosphorylation of eIF2α	0, 2.5, 5, 10, 15, 20, 25, 30, 40 and 50 μM	[161]
	MCF-7, and MDA-MB-231	Induces apoptosis	↓ cancer cell proliferation and augments chemosensitivity of taxol, ↑ cleaved PARP, ↑ Cytochrome c, ↑ Bax, ↓ Bcl-2, ↑ intracellular free calcium concentration	BA - 0.1–50 μMTaxol 0–24 nM	[129]
	MCF7	Induces apoptosis	↓ cancer cell growth, ↑ DNA fragmentation,	IC50 values of 8.32	[162]
	MCF-7	Induces apoptosis	↑ caspase-9 activity, ↑ caspase-3, ↑ Bax, ↑ Bak	0, 1, 5, 10, 20, 50 and 100 µg/ µl	[55]
Ductal	T47D	Induces apoptosis	↓ NQO1 protein, ↑ formation of superoxide, ↑ oxidative stress, ↑ TP53 ↑ CDKN1A genes,	0.1 to 100 μg/mL	[157]
Lung	A549, HepG2and 5RP7	Induces apoptosis	↑ rate of Apoptosis, caused G1 cell cycle arrest, ↑ cleaved caspase 3	IC_50_ values of 207.7, 125.0 and 28.3 μg/mL	[163]
	HKULC2, H1299, and H23	Inhibit metastatic ability	↑ cycle arrest in G1 phase, ↓ migration and invasive potential of cells, ↑ p21, ↑ p53, ↓ CD133, ↓ ALDH, ↓ BCL2, ↓ MCL1, ↓ c-Myc expression, ↓ ABCG1 protein	10 µM of betulinic acid nanoparticles	[164]
	A549	Induces apoptosis	↓ histone H3, ↓ NQO1 protein, ↑ formation of superoxide, ↑ oxidative stress, ↑ TP53 ↑ CDKN1A genes, ↓ p53, ↑ BAX/BCL-2 ratio	0.1 to 100 μg/mL	[157]
	NCI-H460	Antimetastatic and Apoptosis	↑ caspase-3, 6 and 9), ↑ BAX, ↑ BAK, ↓ BCL-2, ↓ p53, ↓ MMP-2/-9. ↓ Osteopontin	10, 25, 50, 75, and 100 µM	[165]
	A549	Induces apoptosis	↑ caspase-9 activity, ↑ caspase-3, ↑ Bax, ↑ Bak	0, 1, 5, 10, 20, 50 and 100 µg/µL	[55]
	A549	Induced apoptosis	↓ PCBP1, ↓ isoform 1 of 3-HAD CoA dehydrogenase, ↓ HSP 90-α 2, ↓ ECH	0, 12.5, 25, 50 and 100 μM	[166]
	A549	Induces apoptosis	↓ proliferation, migration and invasion by tumor cells, ↓ bcl-2, ↑ bax, ↓ cyclin D1, No change in CREB phosphorylation,	0, 2.5, 5, 10 and 25 μM	[46]
Gastric	SNU-16 and NCI-N87	Triggers apoptosis	↑ cytotoxic and inhibitory effects on cancer cells, ↓ migratory and invasive abilities of cancer cells, ↓ EMT progression, ↓ N-cadherin, ↑ E-cadherin	0, 2.5, 5, 10, 20, 40 and 80 μM	[166]
	BGC-823, MNK45 and 293T	Induces apoptosis	↓ proliferation and migration the cancer cells, ↓ expression of VASP mRNA, ↓ Cyclin D1, ↓ PCNA, ↓ c-Myc, ↓ AKT, ↓ Vimentin, ↓ NF-κB activity, ↓ p-p65 protein	0–60 μM	[167]
	SGC7901	Induced apoptosis	↓ cell proliferation, ↑ Caspase- 3 and 9 activities, caspase-8 activity remained unchanged, ↑ PARP cleavage, ↑ Bax,↑ Bak, ↓ Bcl-2, ↓ XIAP, ↑ intracellular ROS level,	0, 1, 5, 10, 20, 50, 100 µg/ µL	[56]
Bladder	T-24, UMUC-3, and 5637	Induced apoptosis	↓ cell proliferation and migration potential of cells, ↓ Cdc25c, loss of mitochondrial membrane potential, ↑ Bax, ↑ cleaved- PARP, ↑ caspase-3, 8, and 9, ↓ wound healing and invasion ability, ↓ Snail, ↓ Slug, ↓ MMP-9	0, 10, 15, 20 and 30 µg/ µL	[168]
Colon	HCT116 and HT29	Induced apoptosis	↓ viability of HCT116 cells, ↑ number of floating cells, ↑ rounding of cells, ↑ emergence of irregular bulges in cell membrane, ↑ condensed chromatin, ↑ micronucleation,	0, 1, 5, 10, 20, 50 and 100 µg/ µL	[169]
	HT-29	Induces apoptosis	↓ proliferation, migration and invasion by tumor cells, ↓ Bcl-2, ↑ Bax, ↓ cyclin D1, No change in CREB phosphorylation, Amounts of the CREB protein, and ERK1/2, Akt, CaMKII kinases remained unchanged	0, 2.5, 5, 10 and 25 μM	[46]
	HCT116, SW480 and DLD-1	Promoted apoptosis and inhibited metastasis	↑ Bax, ↑ caspase-3, ↓ Bcl-2, ↑ ROS, ↓ mitochondrial membrane potential, ↓ migration and invasion of colorectal cancer cells, ↓ MMPs, ↑ MMPs inhibitor (TIMP-2)	0, 05, 10, 20, 40 and 80 μM	[169]
Pancreatic	Mia PaCa-2 and Panc-1	Inhibits stemness	↓ proliferation and tumorsphere formation, ↓ EMT, activates AMPK signaling ↓ mRNA expression levels of Sox2, Oct4, ↓ Nanog and Nanog, ↑ E-cadherin, ↓ vimentin, ↓ effects of gemcitabine on stemness, ↑ sensitivity of pancreatic cancer cells to gemcitabine	0, 12.5, 25, 50, 100 and 200 µM	[170]
Hepatocellular	HepG2, LM3, and MHCC97H	Induces apoptosis	↓ cell viability and proliferation, ↓ migration and invasion, ↓ adhesive ratios, ↑ condensed nuclei and nuclear fragmentation, ↑ apoptosis rate significantly, ↑ Bax, ↑cleaved caspase-3, ↓ Bcl-2, ↓ ROS level, lost mitochondrial membrane potential, ↓ MMP-2 and MMP-9, ↑ TIMP2	2.5–40 μM	[171]
	HepG2	Induces apoptosis	↑ caspase-9 activity, ↑ caspase-3, ↑ Bax, ↑ Bak	0, 1, 5, 10, 20, 50 and 100 µg/µL	[171]
Renal	786-O and ACHN	Induces apoptosis	↓ migrative and invasive capabilities of cancer cells, ↓ Bcl2, ↓ Bcl-2, ↑ Bax, ↑ cleaved caspase-3, ↓ B-cell lymphoma 2, ↑ ROS, ↑ loss of mitochondrial membrane potential, ↓ MMP-2, ↓ MMP9, ↓ Vimentin, ↑ tissue inhibitor of metalloproteinase 2, ↑ E-cadherin	0, 5, 10 and 20 μg/mL	[172]
	786-O and Caki-2	mTor activation	↓ colonies of cancer cells, ↓ glucose consumption, ↓ lactate production, ↓ p-S6, p-4EBP1, ↓ aerobic glycolysis	0, 0.5, 1 and 5 μM	[173]
	RCC4	Induces apoptosis	↓ cell viability, ↑ caspase-3, 7, 8 and 9, ↑ TRAIL R1/DR4 and R2/DR5, ↑ TNFR1, ↑ cytotoxicity, ↑ cleaved PARP, ↓ protein 1 (MDR1), ↑ t-Bid, ↑ Bax, ↑ PuMA, ↓ Bcl-2, ↓ XIAP	0, 6.25, 12.5, 25 and 50 μM	[174]
Neuroblastoma	SK-N-AS	Induces apoptosis	↓ proliferation, migration and invasion by tumor cells, ↓ bcl-2, ↑ bax, ↓ cyclin D1, No change in CREB phosphorylation,	0, 2.5, 5, 10 and 25 μM	[46]
Prostate	LNCaP and PC-3	Induced apoptosis	↓ STAT3 (Y727), ↓ c-Jun (S63), ↓ eNOS (S1177), ↓ ap70 S6 kinase (T389), ↓ p53 (S392) ↓ PYK2 (Y402)	1–90 μM	[175]
Ovarian	SKOV3 and SW626	Inhibited metastasis	↓ proliferation, ↓ N-cadherin, ↑ E-cadherin, ↓ EMT process	0, 2.5, 5, 10, 20, 40, and 80 μM	[176]
	A2780	Induces apoptosis	↓ viability of cancer cells, ↑ condensation of nuclei, ↑ caspase-8, 3,9, ↑ Bax,	25 and 50 µM	[177]
Cervix	HeLa	Suppresses angiogenesis	↓ hypoxia-induced accumulation of HIF-1α,↓ VEGF, ↓ GLUT1, PDK1, ↑ β1, β 2, and β 5 activities of the proteasome	3–30 μM	[178]
	HeLa	Induces apoptosis	↓ cancer cell growth, ↑ nuclear condensation and fragmentation,	IC_50_ values of 6.67	[162]
Equine malignant melanoma	PriFi1, PriFi2, MelDuWi and eRGO1	Induces apoptosis	↓ cell proliferation, ↓ cell viability, ↑cell cycle arrest	--	[179]
Canine osteosarcoma	D-17	Induces apoptosis	↓ Growth of cancer cells. arrested cell cycle in S phase, ↑ %age of apoptotic cells	1, 5, 10, 15, 20, 25, 30 and 40 μM	[47]

**Table 2:** Anticancer effects of betulin and betulinic acid based on in vivo studies.

Type of Cancer	Animal Models	Effects	Mechanisms	Dosage	Duration	References
Oral squamous	Balb/c nude mice injected with KB cells (1 × 10^7^ cells per mouse)	Inhibited the increase in tumor volume	↓ p53 in implanted tumor, ↓ STAT3 signaling, ↓ p- STAT3 in tumor tissues declined	50, 75 and 150 mg/kg	21 days	[156]
Colorectal	BALB/c nude mice xenografted with HCT116 cells (1 × 10^7^ cells per mouse)	Inhibits metastasis	↓ MMP-2, ↓ Ki-67, ↑ caspase-3	0, 10, and 20 mg/kg	21 days	[180]
Gastric	BALB/c nude mice xenografted with SNU-16 cells (1 × 10^7^ cells/mouse)	Delay tumour growth and inhibit pulmonary metastasis	↓ tumour weight, ↓ number of metastatic nodules, ↓ Ki-67 ↓ MMP2	40 mg/kg	21 days	[181]
Breast	Adult orange zebra danio fishes	Anti-angiogenesis	↓tail fin regrowth	Betulin suspension (BetS) (5 mg/g of betulin) and Group III – BeTNS (5 mg/g of betulin)	15 days	[160]
Breast	Balb/c-nu/nu mice subcutaneously injecting MDA-MB-231 cells (5 × 10^6^)	Inhibited tumor growth	↓ Body weight loss, ↑ apoptosis ratio, ↓ Ki67 expression, ↑ expression of GRP78, ↑ CHOP	BA 250 mg/kg + taxol 10 mg/kg	24 days	[129]
Breast	Balb/c nude mice xenografted with MDA-MB-231 cells (2 × 10^5^)	Inhibits metastasis	↓ MMP-2 & 9, ↓ vimentin, ↑ E-cadherin, ↑ GRP78, ↓ *β*-catenin, ↓ c-Myc	125 and 250 mg/kg	28 days	[161]
Hepatocellular	NOD/SCID mice implanted subcutaneously with 100 μL HepG2 cells suspensions (1 × 10^7^ cells/mouse)	Reduces tumour growth	↓ Ki-67 positive cells, ↓ MMP-2 positive cells, ↓ cancer cell proliferation, ↓ Extents of metastatic nodules, ↓ lung weights	10 mg/kg	18 days	[171]
Renal	BALB/c nude mice injected with 786-O cells (1 × 10^6^ cells per mouse)	Inhibits metastasis	↓ Ki67-positive cells, ↓ MMP9-positive cells,	0, 5, and 10 mg/kg	15 days	[172]
Ovarian	BALB/c nude mice injected with SKOV3 cells (5 × 10^6^ cells)	Inhibits tumor growth and Inhibited metastasis	↓ EMT process, ↓ Ki-67+ cells, ↓ MMP-2+ cells	40 mg/kg	21 days	[176]

## Data Availability

Not applicable.

## Abbreviations

hUGT1A3: UDP-glucuronosyltransferase 1A3 (human),hSULT2A1: human dehydroepiandrosterone sulfotransferase, ADME: Absorption Distribution Metabolism Excretion, TPSA: Total prostate-specific antigen, NF-κB: Nuclear factor kappa B, Bcl-2: B-cell lymphoma 2, XIAP: X-linked inhibitor of apoptosis protein, Bak: BCL-2 antagonist/killer, Bax: BCL-2–associated X, IKKα: IκB kinase, ICAM-1: Intercellular adhesion molecule-1, COX: Cyclooxygenase, TNF-α: Tumor necrosis factor α, NADPH: Nicotinamide-adenine dinucleotide phosphate, Keap1: Kelch-like ECH-associated protein 1), ARNT: Aryl Hydrocarbon Receptor Nuclear Translocator, AhR: Aryl hydrocarbon receptor, CYP1A1: Cytochrome P450 Family 1 Subfamily A Member 1, JNK: c-Jun N-terminal kinases, ERK: extracellular signal-regulated kinase, Fas:FS-7-associated surface antigen, VEGF: Vascular endothelial growth factor, IL: Interleukin, IFN-γ: Interferon gamma, iNOS: Inducible nitric oxide synthase, CXCR4:C-X-C chemokine receptor type 4, TRAIL: TNF-related apoptosis-inducing ligand.
